# Accuracy of hands *v*. household measures as portion size estimation aids

**DOI:** 10.1017/jns.2016.22

**Published:** 2016-07-11

**Authors:** Alice A. Gibson, Michelle S. H. Hsu, Anna M. Rangan, Radhika V. Seimon, Crystal M. Y. Lee, Arpita Das, Charles H. Finch, Amanda Sainsbury

**Affiliations:** 1 The Boden Institute of Obesity, Nutrition, Exercise & Eating Disorders, Sydney Medical School, Charles Perkins Centre, The University of Sydney, NSW 2006, Australia; 2 School of Life and Environmental Sciences, Charles Perkins Centre, The University of Sydney, NSW 2006, Australia

**Keywords:** Portion size estimation, Dietary assessment, Food records, Household measures

## Abstract

Accurate estimation of food portion size is critical in dietary studies. Hands are potentially useful as portion size estimation aids; however, their accuracy has not been tested. The aim of the present study was to test the accuracy of a novel portion size estimation method using the width of the fingers as a ‘ruler’ to measure the dimensions of foods (‘finger width method’), as well as fists and thumb or finger tips. These hand measures were also compared with household measures (cups and spoons). A total of sixty-seven participants (70 % female; age 32·7 (sd 13·7) years; BMI 23·2 (sd  3·5) kg/m^2^) attended a 1·5 h session in which they estimated the portion sizes of forty-two pre-weighed foods and liquids. Hand measurements were used in conjunction with geometric formulas to convert estimations to volumes. Volumes determined with hand and household methods were converted to estimated weights using density factors. Estimated weights were compared with true weights, and the percentage difference from the true weight was used to compare accuracy between the hand and household methods. Of geometrically shaped foods and liquids estimated with the finger width method, 80 % were within ±25 % of the true weight of the food, and 13 % were within ±10 %, in contrast to 29 % of those estimated with the household method being within ±25 % of the true weight of the food, and 8 % being within ±10 %. For foods that closely resemble a geometric shape, the finger width method provides a novel and acceptably accurate method of estimating portion size.

Accurate information about the food intake of individuals and populations is notoriously difficult to obtain, is usually reliant upon self-report and can be subject to large errors^(^^1^^,^^2^^)^. There is much debate in the literature about the value of self-reported food intake, particularly for research into energy intake and obesity^(^^3^^,^^4^^)^. However, insight into the types and amounts of foods consumed by individuals or populations is crucial in any investigation about diet and health, for informing food and nutrition policy, as well as being a valuable tool in clinical and education settings^(^^5^^)^.

Food records have been the choice of dietary assessment method for national nutrition surveys in several countries^(^^6^^–^^9^^)^ and are used by clinicians and patients for monitoring adherence to dietary prescriptions in clinical and educational settings, particularly for weight management^(^^10^^–^^12^^)^. Food records involve the individual recording all food consumed in real time (i.e. at the time of consumption) over a defined number of days. Food records can be completed with pen and paper or electronically (e.g. via a website or smartphone application). Weighed food records – where individual foods or ingredients are weighed prior to consumption – are considered precise, but the tedious nature of weighed food records can change intake, and weighing foods is not always feasible^(^^2^^,^^13^^,^^14^^)^. As such, instead of weighed food records, estimated food records are often used, where participants are typically asked to estimate portion sizes in household measures (volumetric cups and spoons) or by describing the size of the portion (‘small’, ‘medium’ or ‘large’). However, the reduction in participant burden with estimated compared with weighed food records is associated with impaired accuracy and considerable error^(^^2^^,^^13^^,^^15^^,^^16^^)^. Given the significant role of food records in investigations of diet and health, as well as the fact that food is frequently consumed away from home without access to scales or other portion size estimation aids, there is clearly a need for a method to estimate portion size that is neither expensive nor burdensome, is flexible for use when eating outside the home, and is relatively accurate.

The use of hands to estimate portion size can potentially fill all of the above criteria. Hands are used by health professionals and the lay public as a guide to portion size^(^^17^^)^. For example, ‘a fist’, ‘thumb tips’ and ‘finger tips’ are used to estimate one cup, one tablespoon and one teaspoon, respectively^(^^18^^,^^19^^)^. However, to our knowledge there have been no studies assessing the accuracy of such hand-based methods. This is particularly important given that hand sizes vary considerably amongst individuals. Additionally, existing hand measures (fists and thumb or finger tips) are merely an alternative to household measures (cups and spoons), and thus have not filled the gap in providing a way to quantify portion sizes of foods that, without access to other portion size estimation aids, may rely on subjective, categorical size estimates (small, medium and large).

To provide a more objective measure of portion size, we developed a novel way to use hands as a portion size estimation aid. Specifically, the width of the fingers was used as a ‘ruler’ to measure the dimensions of foods, and geometric volume formulas and food density factors were subsequently used to estimate weight. The primary aim of this study was to test the accuracy of this novel finger width method to estimate portion size, and to compare this with household measures. We hypothesised that the finger width method would be superior to household measures, due to its comparatively more objective measure of volume and size. A secondary aim was to test the accuracy of fists and tips (thumb and fingers) as portion estimation aids.

## Methods

### Subject recruitment and inclusion criteria

Participants (*n* 67) were staff and students at the University of Sydney, as well as the general public. They were recruited by flyers placed around the campus and on the university website. The study was conducted in accordance with the Declaration of Helsinki and was approved by the University of Sydney Human Research Ethics Committee. To be eligible, participants had to be at least 18 years of age, have no vision impairment and have functional use of both hands. All participants provided informed written consent.

With forty-two food and liquid items to be estimated by all participants, we aimed to recruit at least fifty participants, as this would result in over 2000 observations of the test method, which we reasoned was more than sufficient, given a previous validation study which used a similar number of foods (forty-five foods in the previous study compared with forty-two foods in the current study), but each food was only estimated by between twenty-one and twenty-five people (or approximately 1000 observations)^(^^20^^)^. Due to interest in the study we exceeded our recruitment target.

### Selection and preparation of test foods

A total of twenty-one different foods and liquids, each in two different sizes, were tested (Table 1). The selected foods and liquids were designed to provide a diverse range of weights, shapes, sizes and forms (not only solids and liquids, but also amorphous and semi-solid). The aim was to include foods and liquids that are often ambiguous in estimated food records and for which further clarification is frequently required prior to entry into nutrient analysis software. As such, foods that can be quantified using units (e.g. a slice of bread or ten potato chips) were not included. Foods were pre-cooked (if applicable) and pre-weighed, and were presented to participants on 27 cm diameter white plates. Liquids were presented in different shaped glassware (a champagne flute and a wine glass for wine, and a wide and a tall glass for juice). Honey was placed in a small oval dish, and oil-based spread was placed on the tip of a knife that was placed on a 27 cm diameter white plate. Solid foods and liquids were categorised into one of three geometric shapes they most closely resembled (triangular prism, cylinder or rectangular prism), and were estimated with the finger width method. Amorphous foods and muffins were estimated with the fist method, and semi-solid foods (honey and oil-based spread) were estimated with the tip method.

**Table 1.** Foods and liquids estimated in this study, as well as the density factors, hand method subtypes, geometric shapes and formulae used to calculate volume and estimated weight

Food or liquid item	Density factor (g/ml)*	Hand method subtype and geometric shape	Formula used to convert to volume (ml)†
Soft cheese (Brie)	1·06	Finger width (triangular prism)	(Length × width × height × finger width^3^)/2
Pizza	0·72		
Watermelon	1·00		
Cake	0·50		
Wine	1·05	Finger width (cylinder)	[(π × (Width × finger width/2)^2^) × (height × finger width)]
Juice	1·05		
Sweet potato	0·84		
Meat pie	0·61		
Burger patty	0·94		
Caramel slice	1·14	Finger width (rectangular prism)	(Length × width × height × finger width^3^)
Hard cheese (Feta)	1·00		
Lasagne	1·04		
Fish fillet	1·10		
Chicken breast	1·20		
Beef steak	1·20		
Cereal	0·17	Fist	Number of fists × fist volume
Mashed potato	0·98		
Muffin	0·61		
Rice	0·67		
Honey	1·43	Thumb or finger tip	Thumb tip volume × number of thumb tips; or finger tip volume × number of finger tips
Oil-based spread	0·96		

* From the Australian Food, Supplement and Nutrient Database (AUSNUT) 2012–2013^(^^24^^)^.

† Dimensions (length, width, height) are in number of finger widths, and finger widths are in cm.

Although we had the true weight of the foods as the reference against which to assess accuracy of the hand method, we included household measures (cups and spoons) and subjective, categorical size estimates (small, medium and large) as additional reference estimation methods, because these are used in situations where people do not have access to scales or other portion size estimation aids. Indeed, in the UK Women's Cohort Study, one of the largest cohort studies investigating associations between diet and cancer in the UK^(^^21^^)^, participants received the following written instructions in their food record: ‘If you do not have scales at home, or if you are eating food away from the home, then describe the food you eat using household measures e.g. tablespoons, cups, large glass etc.’^(^^22^^)^. Categorical size estimations have also been used in quantitative FFQ^(^^23^^)^. Due to the inclusion of participants from a wide range of ages, BMI and from both sexes, as well as the lack of disaggregated Australian-specific median food portion sizes, we did not assign weights to the categorical size estimations.

### Procedure

**Fig. 1.**
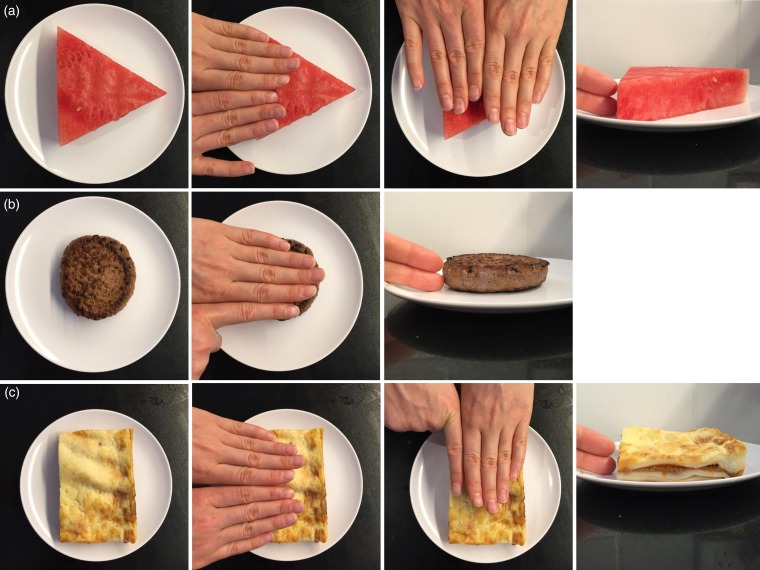
Demonstration of the finger width method for geometrically shaped foods and liquids. This method used the width of the fingers as a ruler to measure the dimensions of a food or liquid. Foods or liquids estimated with this method were categorised into one of the three geometric shapes they most closely resembled: (a) triangular prism, (b) cylinder or (c) rectangular prism. For triangular and rectangular prisms, the length, width and height of the foods were measured, and for foods that resembled a cylinder, the diameter and height of the foods were measured. The number of finger widths for any given dimension was then converted to cm using the average width of one finger. Using geometric formulas of the respective shapes, the dimensions of the foods or liquids were then converted to volumes, which were in turn multiplied by density factors to calculate an estimated weight.

The study was conducted over 5 d between 20 January and 2 February 2015. Upon arrival at the clinic, groups of two to four participants at a time were taken through the study procedures. The procedure consisted of five stages, all of which were conducted on the same day and took approximately 1·5 h to complete:

(1) *Household and size method explanation and practice.* In the practice room (separate from the testing room), sets of volumetric household measuring cups (one cup, ¾ cup, ½ cup, ⅓ cup, ¼ cup) and spoons (one tablespoon, ½ tablespoon, one teaspoon, ½ teaspoon) were displayed on a table along with seven practice food and liquid items (beef steak, quiche, glass of milk, lemon tart, slice of cake, plain spaghetti and peanut butter). Participants were given a verbal explanation and demonstration of how to estimate portion sizes using household measures as an aid, and were asked to estimate and record the volume (in cups or spoons) of each food or liquid, using any number including fractions or increments of whole numbers, e.g. 1/5 cup or 2·5 cups). Participants were told that cups and spoons would not be provided during the testing stage, to reflect real-world situations when estimating portion sizes away from home. They were also asked to indicate the size (small, medium or large) of all of the food and liquid items. Participants were encouraged to seek assistance or ask questions during the practice session if they were unsure about what to do.
(2) *Household and size method testing*. Participants were taken into the testing room where forty-two test foods and liquids were presented separately in test stations around one long table (the order of which had been randomly determined). Participants were instructed which station to start at, leaving an approximately even space between participants, and to estimate portion size at that test station using household measures and subjective size estimation and to record their estimations on the data entry sheet, before moving onto the next test station, moving in a clockwise direction around the table. They were instructed to not compare foods between test stations or on their data entry sheet, which was checked for completeness prior to moving on to the next stage.
(3) *Hand method explanation and practice*. Participants were provided with an explanation of each of the hand methods, namely finger width, fist, and tips (thumb or index finger):
  (a) *Finger width*. Participants were asked to measure the dimensions of food items using the width of their fingers (from the first joint of the little finger to the second joint of the index finger) as a ‘ruler’. This procedure was demonstrated as shown in Fig. 1. For any one dimension they could use a fraction of a finger (e.g. ½ a finger) or any number of fingers they needed to take the measurement when all fingers were side by side. For example, if more than eight fingers were needed, they would have to cross their arms and use the first hand again. Participants were instructed to measure the food at the longest, widest or highest point.
  (b) *Fist*. For the fist method, participants were instructed to compare the food against the volume of their fist up until the wrist joint, and to estimate the volume of the food in a multiple of the number of fists. They were reminded that the volume of the hand does not change whether it is flat or clenched into a fist, and as such they could mould their hand into an alternate shape to help them estimate the volume of the food. Similar to the household method, they were told that they could indicate in fractions of a whole or greater than a whole fist.
  (c) *Tip*. For the tip method, participants were instructed to compare the food against the volume of the tip of their left or right thumb (up until the first joint or crease) or index finger and to indicate how many ‘tips’ they thought the food was equivalent to. Thumb and finger tips were indicated as separate options on the data entry sheet for participants to select the more suitable option for the food.
(4) *Hand method testing*. As per stage 2, except that participants were provided with a data entry sheet that was specific to the hand methods. The data entry sheet prompted participants on what dimensions to measure (e.g. length, width and height for foods that resembled a rectangular or triangular prism in shape, and diameter and height for foods in the shape of a cylinder) (Table 1).
(5) *Anthropometric and hand measurements*. Upon completion of both estimation methods, participants were called individually into a private interview room, where their age and sex were recorded and their height, weight and hand measurements were measured and recorded. Height was measured with a stadiometer (SECA model 220 Telescopic Height Rod; SECA) to the nearest 0·1 cm with the participant looking straight ahead and without shoes. Weight was taken in light clothing and without shoes to the nearest 0·1 kg (Tanita WB-100A; Tanita Corporation of America, Inc.). The width of one finger was calculated from the average width of four fingers from the non-dominant hand, measured using vernier calipers between the first joint of the little finger (distal interphalangeal joint) and the second joint of the index finger (proximal interphalangeal joint), with the hand placed flat on a table, palm side down. Fist volume was measured by weighing the water displaced from a beaker when the fist was submerged up to the base of the palm. Tip (thumb tip and fingertip) volumes were measured by wrapping the whole thumb or finger in plastic film and inserting up to the first crease into a jar of Play-Doh® (Hasbro Australia Ltd). Volume was calculated from the volume of water required to fill the resultant indentation. All hand measures were taken in duplicate and repeated if there was greater than 10 % discrepancy between the two measures.

### Data processing and analysis

Data were entered into Microsoft Excel from the paper forms by one author (M. S. H. H.), and all data entry was checked for accuracy by another author (A. A. G.). Raw data were converted to an estimated volume using the equations shown in Table 1. Estimated volumes were then converted to weights using the food density factors also shown in Table 1. Density factors were taken from the closest match of the food item from the Food Measures Database in the most recent version of the Australian Food, Supplement and Nutrient Database (AUSTNUT) 2011–13^(^^24^^)^.

### Statistics

Data on participant characteristics and hand measurements are presented as mean values and standard deviations and ranges. A two-sample *t* test was used to compare participant characteristics and hand measurements between females and males. All other data were non-parametric and are presented as medians and interquartile ranges. Wilcoxon signed-rank tests were used to compare the true weight of each food item with the weight estimated with the hand method and the household method. The Bonferroni correction was applied to correct for multiple comparisons. The corrected significance level was *P* < 0·001. Accuracy was calculated as the percentage difference between the estimated weight and the true weight of the food, using the following equation: ((estimated weight − true weight)/true weight) × 100. Thus, a positive value indicates an overestimation and a negative value indicates an underestimation of the true weight of the food. The median percentage difference was then graphed to visually compare the accuracy between each of the hand method subtypes with the household method. The number of food or liquid items in which the median estimation error fell within ±50 %, ±25 % and ±10 % of the true weight was calculated^(^^20^^)^. For the size method, the proportion of participants indicating small, medium and large was calculated. All statistical analyses were performed using SPSS version 22.0 (IBM Corp.).

## Results

### Sample characteristics

Characteristics and hand measures of the sixty-seven participants are shown in Table 2. All hand measures were significantly smaller for females than males. Interestingly, while fist volume of males and females overall was almost exactly one cup (250 ml), consistent with the popular notion that a fist represents one cup, male fists were significantly greater in volume than female fists by approximately 100 ml (*P* < 0·001). Additionally, a thumb tip overall was just slightly less than the volume of a teaspoon (5 ml), despite often – albeit not always – being used as an estimate of a tablespoon in public portion estimation recommendations.

**Table 2.** Participant characteristics and hand measurements† (Mean values, standard deviations and ranges)

	Overall			Males			Females		
Measurement	Mean	sd	Range	Mean	sd	Range	Mean	sd	Range
*n*	67			20			47		
BMI (kg/m^2^)	23·2	3·5	17·9–30·8	25·6	3·8	17·9–30·8	22·2	3·8	18–29·7
Age (years)	32·7	13·7	19–77	36·9	16·1	20–77	31·0	12·3	19–63
Finger width (cm)	1·6	0·2	1·3–2·3	1·8	0·2	1·6–2·3	1·5*	0·1	1·3–1·8
Fist volume (ml)	248·7	57·5	156·3–396·6	321·1	43·0	238·0–396·6	217·9*	27·1	156·3–279·4
Thumb tip volume (ml)	4·8	1·6	2·4–10·9	6·4	1·8	4·0–10·9	4·1*	0·9	2·4–6·4
Index fingertip volume (ml)	2·8	1·0	1·3–6·7	3·7	1·1	2·6–6·6	2·4*	0·7	1·3–4·2

* Mean value was significantly different from that for males (*P* < 0·001).

† The two-sample *t* test was used to compare females and males.

### Geometrically shaped foods and liquids estimated with the finger width method, household method (cups) and size descriptions

Geometrically shaped foods and liquids (*n* 30) were estimated with the finger width method, as well as with the household method (cups) and categorical size descriptors (Table 3). When the estimated weights were compared with true weights, there were eleven items for the finger width method and only three for the household method for which the estimated weight was not significantly different from the true weight. Foods with estimated weights that were significantly different from their true weight tended to be overestimated for both methods (Table 3).

**Table 3.** True weight and weight estimated with each of the hand method subtypes (finger width, fist and thumb or finger tips) and household method (cups)* (Medians and interquartile ranges (IQR))

			Estimated weight (g)					
			Hand			Household		
Food or liquid item	Portion size†	True weight (g)	Median	IQR	*P*	Median	IQR	*P*
Geometrically shaped solid foods or liquids (finger widths)‡								
Δ Soft cheese (Brie)	(1)	9·2	7·6	5·6	0·154	11·3	5·3	<0·001
	(2)	33·3	31·4	13·2	0·461	31·8	8·9	0·023
Δ Pizza	(1)	47·5	41·2	22·3	0·002	90·0	30·0	<0·001
	(2)	83·9	71·5	24·8	<0·001	135·0	90·0	<0·001
Δ Watermelon	(1)	159·6	135·4	41·0	0·009	262·5	65·6	<0·001
	(2)	203·7	166·1	126·5	0·103	262·5	65·6	<0·001
Δ Cake	(1)	64·3	49·4	25·6	<0·001	83·3	31·3	<0·001
	(2)	73·7	64·2	32·8	0·081	93·8	31·3	<0·001
○ Wine	(1)	128·7	148·2	36·8	<0·001	187·5	125	<0·001
	(2)	177·2	215·7	73·4	<0·001	250·0	62·5	<0·001
○ Juice	(1)	140·7	171·7	76·9	<0·001	195·0	65·0	<0·001
	(2)	233·3	275·7	117·1	<0·001	260·0	86·7	<0·001
○ Sweet potato	(1)	32·8	37·5	24·4	0·003	52·5	0·0	<0·001
	(2)	61·2	51·9	28·8	0·004	70·0	52·5	<0·001
○ Meat pie	(1)	48·6	65·2	30·7	<0·001	65·8	49·4	<0·001
	(2)	175·6	250·0	94·3	<0·001	296·3	148·1	<0·001
○ Burger patty	(1)	115·3	98·8	56·8	0·004	150·0	100·0	<0·001
	(2)	231·4	189·7	68·1	<0·001	300·0	100·0	<0·001
□ Caramel slice	(1)	58·7	80·2	35·3	<0·001	71·3	71·3	<0·001
	(2)	137·8	154·3	82·0	<0·001	213·8	142·5	<0·001
□ Hard cheese (Feta)	(1)	13·2	14·8	10·7	0·009	15·0	5·0	0·358
	(2)	15·9	16·6	11·5	0·156	15·0	13·1	0·046
□ Lasagne	(1)	167·1	210·6	100·0	<0·001	260·0	65·0	<0·001
	(2)	350·1	427·2	184·4	<0·001	520·0	130·0	<0·001
□ Fish fillet	(1)	104·8	166·0	100·5	<0·001	275·0	68·8	<0·001
	(2)	165·2	283·2	95·3	<0·001	275·0	91·7	<0·001
□ Chicken breast	(1)	107·8	194·1	81·6	<0·001	200·0	75·0	<0·001
	(2)	195·4	402·2	177·6	<0·001	400·0	300·0	<0·001
□ Beef steak	(1)	54·5	94·4	53·6	<0·001	150·0	75·0	<0·001
	(2)	253·4	485·8	213·2	<0·001	450·0	200·0	<0·001
Amorphous foods and muffins (fists)								
Cereal flakes	(1)	30·4	64·0	30·6	<0·001	31·9	21·3	0·006
	(2)	79	113·4	35·3	<0·001	85·0	21·3	0·773
Mashed potato	(1)	118·7	201·9	64·9	<0·001	183·8	61·3	<0·001
	(2)	199·4	320·6	101·4	<0·001	306·3	122·5	<0·001
Muffin	(1)	18·6	36·7	17·9	<0·001	30·6	0·0	<0·001
	(2)	154·6	184·2	76	<0·001	190·6	76·3	<0·001
Rice	(1)	55·2	107·1	57·9	<0·001	83·8	41·9	<0·001
	(2)	171·5	274·7	88	<0·001	223·3	111·7	<0·001
Spreads (thumb or finger tips)								
Honey	(1)	5·4	15·7	14·5	<0·001	21·5	10·7	<0·001
	(2)	12·9	30·9	22·2	<0·001	42·9	32·2	<0·001
Oil-based spread	(1)	1·1	2·4	1·3	<0·001	2·4	2·4	<0·001
	(2)	13·5	12·0	5·6	<0·001	14·4	7·2	<0·001

* The Wilcoxon signed-rank test was used to compare estimated weight with true weight. The Bonferroni corrected significance level of *P* < 0·001 was used to account for multiple comparisons.

† (1) Indicates the smaller and (2) indicates the larger of the two portion sizes for each item estimated.

‡ Symbols to the left of each food or liquid item indicate the geometrical shape that was used to calculate volume from finger width measurements: Δ, triangular prism; ○, cylinder; □, rectangular prism.

**Fig. 2.**
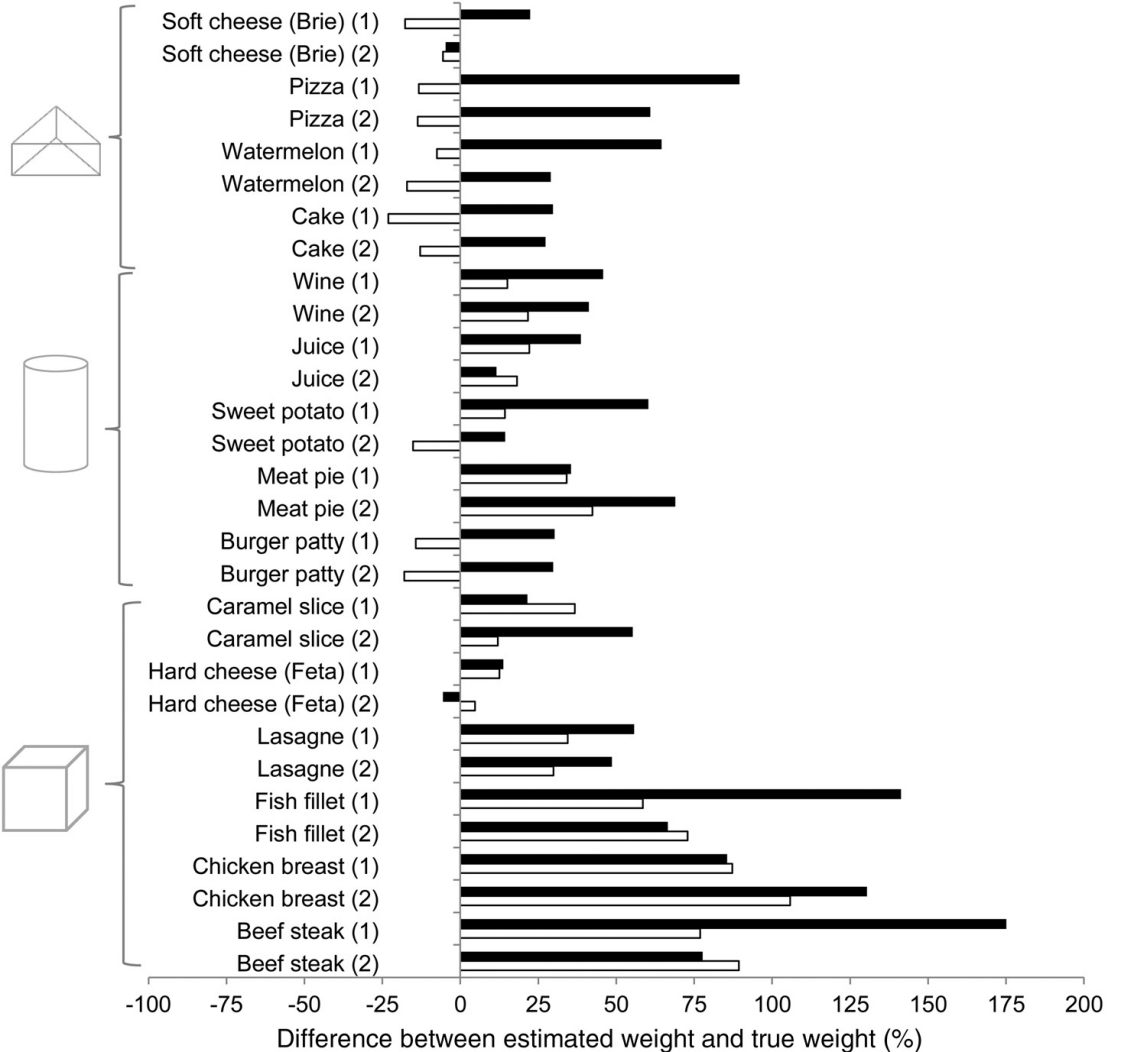
Comparison of portions estimated with the finger width method (□) and household method (cups) (■) with respect to the median percentage difference between the estimated and true weights of geometrically shaped foods and liquids, each in two different sizes. (1) Indicates the smaller and (2) the larger of the two portion sizes for each item estimated.

As the true weights of the foods and liquids ranged from 9·2 g for soft cheese (Brie) to 253·4 g for beef steak, the median percentage difference from the true weight shown in Fig. 2 allows for a more standardised comparison between the different foods and liquids and methods. Foods and liquids that closely resembled the geometric shape used to calculate volume with the finger width method tended to be more accurately estimated than those that did not ‘fill’ or conform to the geometric shape. For example, a slice of pizza or cake generally closely resembles a triangular prism, a glass of juice or burger patty generally closely resembles a cylinder, and a piece of hard cheese such as Feta generally closely resembles a rectangular prism, and these were all well estimated using the finger width method. In contrast, fish fillets, chicken breasts and beef steaks are generally more irregular than a rectangular prism, and for all of these three foods in either size (the six items at the bottom of Fig. 2), estimations with both the finger width method and the household method were all above 50 % of the true weight. For the remaining twenty-four foods and liquids (the twenty-four items at the top of Fig. 2), all were within 50 % of the true weight – nineteen of these were within 25 % of the true weight and three were within 10 % of the true weight – when estimated with the finger width method. In contrast, only seventeen of these twenty-four foods and liquids were within 50 %, seven were within 25 % of the true weight and two were within 10 % of the true weight when estimated with the household method (Fig. 2).

For the categorical size method (i.e. when participants were asked to rate the food as small, medium or large), there were no geometrically shaped foods for which 100 % of participants agreed on the size (Table 3). There were only eight geometrically shaped foods, where ≥80 % of participants rated the food as being of the same size (Table 3). For the fish fillet, chicken breast and beef steak (in both sizes) that were not estimated well with either the finger width or the household method, as mentioned above, the size method did not provide a good alternative (Table 3). For instance, for the larger size of the fish fillet and the smaller size of the chicken breast, there was an almost 50:50 split between the proportion of participants that rated the portion size as medium and large and small and medium, respectively. However, for the smaller and larger portions of beef steak, which perhaps represent more extremes on the portion size continuum, ≥80 % of participants rated the food as being of the same size for both sizes. Therefore, categorical size estimates of portion sizes are highly subjective, in that the same portion of food was perceived as being of a different size by different people.

### Amorphous foods and muffins estimated with the fist method, household method (cups) and size descriptions

Amorphous foods and muffins (*n* 8) were estimated with the fist method, as well as with the household method (cups) (Table 3) and categorical size descriptors (Table 4). Cereal flakes (in both sizes) estimated with the household method were the only amorphous foods in which there was no significant difference between the estimated weight and true weight (Table 3). Foods with estimated weights that were significantly different from their true weight were all overestimated for both methods (Table 3). There were only two food items in this category for which ≥80 % of participants rated the food as being of the same size (the smaller muffin and the smaller portion of rice; Table 4).

**Table 4.** Proportion of participants (*n* 67) classifying the food or liquid items within each categorical size category

Food or liquid item	Portion size*	True weight (g)	Small (%)	Medium (%)	Large (%)
Solid foods or liquids					
Soft cheese (Brie)	(1)	9·2	90	9	2
	(2)	33·3	21	55	24
Pizza	(1)	47·5	90	10	0
	(2)	83·9	24	67	9
Watermelon	(1)	159·6	21	60	19
	(2)	203·7	18	66	16
Cake	(1)	64·3	27	69	5
	(2)	73·7	10	72	18
Wine	(1)	128·7	16	60	24
	(2)	177·2	9	56	35
Juice	(1)	140·7	67	33	0
	(2)	233·3	3	70	27
Sweet potato	(1)	32·8	97	3	0
	(2)	61·2	57	36	8
Meat pie	(1)	48·6	98	2	0
	(2)	175·6	2	62	37
Burger patty	(1)	115·3	25	67	8
	(2)	231·4	0	10	90
Caramel slice	(1)	58·7	58	42	0
	(2)	137·8	2	36	63
Hard cheese (Feta)	(1)	13·2	73	21	6
	(2)	15·9	57	30	13
Lasagne	(1)	167·1	28	66	6
	(2)	350·1	2	10	88
Fish fillet	(1)	104·8	12	76	12
	(2)	165·2	2	51	48
Chicken breast	(1)	107·8	43	57	0
	(2)	195·4	3	19	78
Beef steak	(1)	54·5	93	8	0
	(2)	253·4	2	10	88
Amorphous foods and muffins					
Cereal flakes	(1)	30·4	46	52	2
	(2)	79·0	2	24	75
Mashed potato	(1)	118·7	46	51	3
	(2)	199·4	3	25	72
Muffin	(1)	18·6	100	0	0
	(2)	154·6	3	39	58
Rice	(1)	55·2	88	12	0
	(2)	171·5	6	55	39
Spreads					
Honey	(1)	5·4	49	43	8
	(2)	12·9	9	33	58
Oil-based spread	(1)	1·1	96	5	0
	(2)	13·5	5	19	76

* (1) Indicates the smaller and (2) indicates the larger of the two portion sizes for each item estimated.

**Fig. 3.**
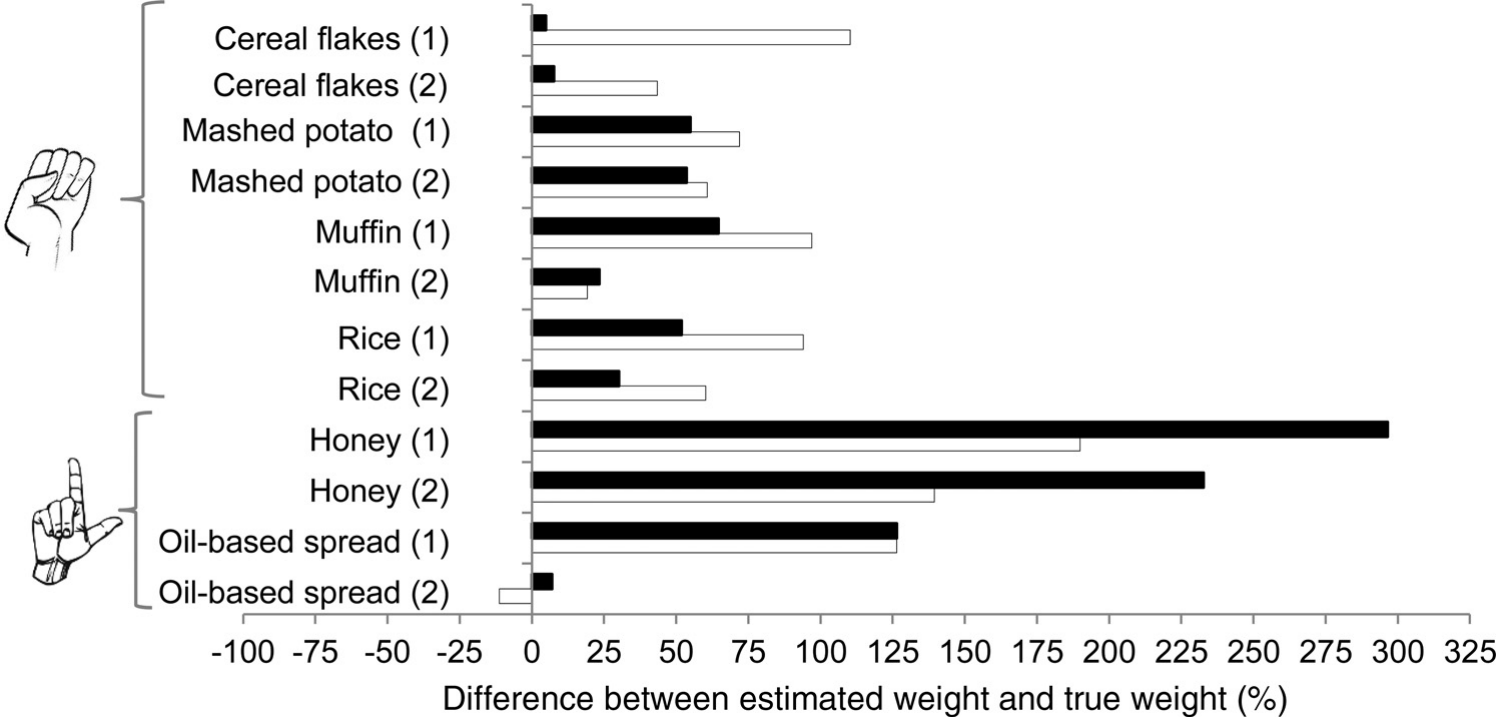
Comparison of portions estimated with the fist and tip (thumb or finger) method (□) and household method (spoons) (■) with respect to the median percentage difference between the estimated and true weights of amorphous foods, muffins and spreads, each in two different sizes. (1) Indicates the smaller and (2) the larger of the two portion sizes for each item estimated.

The median percentage difference between the estimated and true weight of amorphous foods and muffins is shown at the top of Fig. 3. For the fist method, no food was estimated to within 10 % of its true weight, only one food was estimated to within 25 % of its true weight (the larger muffin), one food was estimated to within 50 % of its true weight (the larger portion of cereal flakes), and for all of the remaining six foods, estimations were greater than 50 % of the true weight. For the household method, three foods were estimated to within 10 % of their true weight (both sized portions of the cereal flakes and the larger oil-based spread), one food was within 25 % of the true weight (larger muffin), and one food was estimated to within 50 % (larger size of the rice) of its true weight, and for all of the remaining four foods, estimations were greater than 50 % of the true weight.

### Spreads estimated with the tip method, household method (spoons) and size descriptions

Spreads (*n* 4) were estimated with the tip (thumb or finger) method, household method (tablespoons or teaspoons) and size descriptions (Table 3). The estimated weight of all four of these test food items was significantly different from their true weights, when estimated with either the hand or the household method (Table 3). Only the small portion of the oil-based spread resulted in ≥80 % of participants rating the food as being of the same size, highlighting that spreads – like other foods and liquids – are not well estimated with size descriptions, either.

The bottom section of Fig. 3 shows the median percentage difference from the true weight for spreads estimated with both the tip (thumb or finger) method and the household method. Only the larger portion of the oil-based spread was within 25 % accuracy for both methods (Fig. 3).

## Discussion

Individuals have difficulty in accurately estimating portion sizes using a variety of methods. The primary aim of this study was to assess the accuracy of estimating portion sizes using the width of the fingers as a ‘ruler’ to measure the dimensions of foods and liquids that resemble geometric shapes (triangular prisms, cylinders and rectangular prisms). While there was a significant difference between the estimated weight and true weight of 65 % of the thirty food items estimated with the finger width method, our findings were in line with our hypothesis that this method would be superior to household measures or size descriptions, due to its comparatively more objective measure of volume and size. Indeed, with the exception of both sizes of fish fillets, chicken breasts and beef steaks, nineteen of the remaining twenty-four foods and liquids estimated with the finger width method (80 %) were found to be within ±25 % of the true weight of the food or liquid (and 100 % were within 50 % of the true weight), compared with only seven of those estimated with the household method (29 %, and 34 % being within 50 % of the true weight). Our results are comparable with those from a previous study investigating the use of food photographs to estimate portion size, which found that 83 % of the 135 food portion estimations for forty-two food and liquid items had mean estimations within 25 % of their true weight^(^^20^^)^. The advantage of the finger width method over food photographs, however, is that fingers but not photographs are always available to people as a tool for portion size estimation. To put a 25 % estimation error in context, this equates to approximately one large mouthful of juice or two large sips of wine from a portion of 150 ml, for example.

While the finger width method provided a reasonably accurate method of portion size estimation for foods that closely reassembled geometric shapes, it was not ideal in its current format for estimating foods that did not conform well to a geometric shape (e.g. fish fillets, chicken breasts and beef steaks). There are several possible sources of error and potential solutions that could improve the accuracy of the finger width method for irregularly shaped foods. The largest source of error is most likely the assumptions involved in the use of the geometric formulas used to convert the dimensions into an estimated volume, combined with the fact that participants were asked to measure the widest, longest or thickest point of the food. Despite having fairly uniform dimensions for at least one of the three dimensions measured (length, width or height), the fish fillets, chicken breasts or beef steaks were narrower at one or more points. Thus the food did not conform or ‘fill’ the three-dimensional space created by the rectangular prism calculated from these dimensions, causing overestimations of the volumes and thus weights of the foods. A potential solution may be to ask participants to take an average dimension (e.g. if the dimension was between two and four finger widths, it would be recorded as three finger widths). Another possible source of error with the finger width method is the precision of the fingers for measuring dimensions. Despite providing participants with instructions to estimate using fractions of fingers, the majority of participants chose to estimate the height (thickness) of foods in increments of whole fingers. Consequently, if the thickness of a fish fillet, chicken breast or beef steak was less than or greater than a whole number of fingers thick, this would have introduced large error. For example, the height of the small fish fillet was 0·7 cm (or approximately half the average finger width), yet over half (58 % or 39/67) of the participants reported the height as one finger width. This suggests that in order to improve the accuracy of this method, instructions provided to participants may need to place greater emphasis on the importance of using fractions of a finger, perhaps with the use of examples to demonstrate how it can make an impact on the accuracy of estimations. Alternatively, participants could be provided with a ruler to measure the dimensions of a food, which has greater precision, is portable, cheap and universal and has been used in food recalls^(^^25^^)^. The accuracy of estimating irregularly shaped cuts of meat with rulers has been studied previously^(^^26^^)^. In that study, participants were asked to estimate the length, width and thickness of different cuts of fish, chicken and meat using a ruler or size grid, after the food was removed from sight. Mean estimation errors for length and width were generally within −10 % of the actual dimension; however, thickness was consistently overestimated, by an average of 30–40 %^(^^26^^)^. Therefore, the accuracy may have been higher had the participants used the ruler to measure the food directly, while it was still in sight. However, the benefit of using finger widths over a ruler (or other two-dimensional or three-dimensional portion size aids) is that a person does not have to remember to carry anything with them in order to estimate portion sizes, and it is potentially also more socially acceptable than bringing out a ruler. In summary, possible sources of errors may be attenuated with future modifications to the finger width method for foods that do not conform closely to a geometric shape.

A strength of this study is that in addition to the ‘gold standard’ reference method of weighing, we compared the hand methods against two additional reference methods: household measures and subjective categorical size descriptions. Fewer than a third of geometrically shaped foods estimated with household measures were within 25 % accuracy. One might argue that this finding is not surprising given that for many of these foods, household measures may not be a ‘good’ comparison. For example, a slice of pizza is not usually estimated in ‘cups’. However, our finding highlights that when food is consumed away from home without access to scales or other portion size estimation aids, for many geometrically shaped foods, there was previously no ‘good’ way to estimate portion size other than to use subjective descriptions of size (hence the need for our finger width method). As we have demonstrated here, subjective size categories are not a good alternative to household measures, as there was little consensus (except for portions which perhaps represented more extremes on the portion size continuum) amongst participants as to whether food portions were small, medium or large in size, even though all participants were presented with the same portion sizes. A previous study^(^^27^^)^ investigated the accuracy of a ruler and a two-dimensional adjustable wedge to estimate the length and width of wedge-shaped foods after they had been removed from sight. On average, the total area of pizza was underestimated by 20 %, and that of cake was overestimated by 20 %, which is similar to the error of volumes in our present findings. However, in this study they did not measure the thickness (or height) or the wedge-shaped foods, so a comparison of error in volume (and accordingly, weight) cannot not be made between that study and our present work. Thus, notwithstanding the drawbacks associated with the volumetric formulas as discussed above for foods that do not conform to a geometric shape, having measurements of the dimensions of a food or liquid through the use of finger widths to estimate the size of a portion provides a more objective and reproducible method for coding a portion size compared with household measures or subjective size descriptions.

A secondary aim of this study was to assess the accuracy of fists and tips (thumbs and fingers) as portion size estimation aids. Despite the average volume of a fist being approximately one cup, as well as their potentially greater usefulness than household measuring cups due to their malleability and convenience, amorphous foods were not as well estimated with the fist method as with the household method. It is possible that participants find it easier and more intuitive to visualise ‘filling’ an empty cup with the food when making volume estimations, rather than comparing the food with their fist, which is already ‘filled’. Household cups and spoons are also easier to code into dietetics software for analysis, as measuring fist and finger or thumb tip volume using water is quite arduous and not feasible in many situations. Household measuring cups are also of a standardised and known volume compared with other household items such as tennis balls or golf balls that may be used to guide portion size, the putative volume of which has been shown to be quite erroneous^(^^28^^)^. However, despite the household method (cups) producing slightly less error overall than the fist method for the smaller muffin, mashed potato and rice portions, the median difference between the estimated and true weights of foods was still around 50 %. Our finding that individuals are not good at subjectively assessing volume is in line with the findings of a previous study that compared computer-calculated food volumes from photographs taken from a chest-worn camera device and those subjectively estimated by three volunteers (including one who was a dietitian) with the actual volume calculated through displacement for 100 food items^(^^29^^)^. The computer-calculated volumes were with ±30 % of the true volume of 85 % of the food items, compared with 15–57 % of the food items estimated by the volunteers. Thus, this study shows the difficulty that people have in making subjective volume estimations and that the accuracy between people can be quite large. In summary, neither the household method nor the fist method consistently estimated the portion sizes of amorphous foods or muffins satisfactorily, and their use in estimating portion sizes should be interpreted with caution.

Dietary assessment research has increasingly focused on harnessing technology in an attempt to reduce cost and burden to researchers and participants^(^^11^^,^^30^^)^, and the finger width method could complement these aims. One example is with electronic food records in which data entry is completed by the participant. However, whilst technological advances may help to reduce participant and researcher burden by avoiding duplication of data entry, dietary assessment methods are still subject to the same types of measurement errors, including errors with quantifying portion size. The finger width method could easily be incorporated into electronic food records to improve the accuracy of portion size estimations by reducing the variability of subjective estimation methods. For instance, finger width measurements would be entered along with height and weight, allowing the calculation of portion size to be automated. This would not only be relevant to research, but could also be useful in clinical and educational settings, where the aim is to provide guidance on portion sizes in relation to a clinical objective (e.g. weight loss). However, the use of such an application of the finger width method would be limited to those who own or know how to operate such devices. On the other hand, the finger width method can also be used – as in the present study – as a pen-and-paper method, which makes it portable, inexpensive and universal. These factors would make it suitable for field studies across different population groups, including ethnic minority groups, for people with low literacy, or in contexts where technology is not an option.

Our study design was intended to replicate real-world situations where participants complete food records in real time (and therefore do not rely on memory) and without access to other portion size estimation aids. The generalisability of our findings to different populations, settings and dietary assessment methods is limited by our sample and study design. Whilst we did not impose restrictive inclusion criteria, and we recruited people with a range of BMI and ages, our sample was predominantly female, younger and neither overweight nor obese. Our participants were predominantly university staff and students, and were thus likely to be well educated. We also did not assess prior experience with the use of portion size estimation aids, which could have influenced our results. Further, the training used in this study was brief, and more intensive training, perhaps with the use of food models of known volumes and sizes, could have increase the accuracy of estimations^(^^31^^–^^33^^)^. Additionally, the wide age range of participants included in this study may have contributed to the large variability in responses to the categorical size estimations. For example, differences in metabolic needs, cognitive processing capabilities or prior exposure to different portion sizes between younger and older participants may have increased or decreased estimation errors of particular foods or liquids. Our study design could have also been improved by using dividers to ensure that participants did not consciously or unconsciously compare between foods and liquids when making estimations. Future research should investigate the use of hand methods in a wider range of foods and liquids, as well as retrospective portion sizes estimation, such as for use in dietary recall interviews and in comparison with other types of two-dimensional and three-dimensional portion size estimation aids (e.g. food photographs).

In conclusion, portion size estimations for amorphous foods, using hand or household measures, should be interpreted with caution due to the considerable error associated with these techniques for these food types. In contrast, for foods or liquids that conform to a geometric shape, the finger width method may be used to estimate portion size instead of household measures or subjective categorical size descriptions. Thus, as the finger width method is not suitable for all foods, it should be seen as a complementary, rather than a stand-alone portion size estimation method. However, this reflects the fact that food and liquid portion size estimation requires the use of a combination of methods to match the various shapes and physical forms of foods. The finger width method offers advantages over other options because it is portable, inexpensive and universal, which would make it suitable for studies in a wide variety of people and contexts, including limited access to technology. However, before the finger width method can be used more generally, further validation studies incorporating a wider range of foods and in specific population groups (e.g. younger, older, lower education levels) should be conducted. Future studies should also investigate ways to modify the finger width method to better estimate more irregularly shaped foods such as fish fillets, chicken breasts and beef steaks.
